# Ramadan: devotion, compassion, and purification in Sydney

**DOI:** 10.1007/s11562-022-00497-3

**Published:** 2022-11-18

**Authors:** Adam Possamai, Kathleen Openshaw, Pedram Khosronejad, Ayesha Rasheed, Aisha Mubashar

**Affiliations:** Western Sydney University, Sydney, Australia

**Keywords:** Ramadan, Sydney, Representations of Islam, Islam, (Social) media

## Abstract

While Ramadan in Western societies has been studied extensively in relation to health issues, no research to date has explored its representation through social scientific lenses. This article uses the Greater Western Sydney region in New South Wales, Australia, as a case study. This agglomeration of suburbs from the outer western suburbs of Sydney to the Blue Mountains has the highest proportion of Muslims in the country. To understand the representation of Islam in this region, this paper first analyses the articles in its major and local newspapers to then contrast them to the way the Ramadan festival is represented by mosques on their websites. This research discovers that Ramadan in Sydney newspapers tends to be reported in a secular fashion with a stronger focus on its public and economic activities. The focus of a large proportion of these articles on the way it attracts business demonstrates that it is a well-accepted event in Australia. In contrast, the pictures provided in Muslim sites in Sydney are more religious than the newspaper depiction and show a contrast with regard to ethnicity and gender. While the newspaper pictures are from the public sphere and tend to be multicultural across various Muslim ethnicities and do not show gender segregation, the online pictures from Muslim organisation show a strong gender segregation and represent the local ethic community they serve. While the representations in the public sphere are neo-liberal post-secularism and multiculturalism, those from these organisations are curating religiously important rather than business orientated moments in their community.

## Introduction

Ramadan is the ninth month of the Islamic calendar and is considered the holiest month of the year, during which time Muslims across the world fast, abstaining from food and drink, from sunrise to sunset. Ramadan is one of the five pillars of Islam and is understood as an obligation upon all Muslims who are in good health. Ramadan is considered by Muslims to be a time for self-examination, increased religious devotion, and an opportunity to broaden one’s compassion for the less fortunate and for community engagement. Through this temporary deprivation, Muslims renew their awareness of, and gratitude for, everything God has provided in their lives.

In Western countries, where there is very little literature about how Ramadan specifically is celebrated in the West, how is this religious event practised by local Muslims? What community groups and religious institutions are involved in organising the events during this holy month? How is this practice received and perceived by the larger community -*Ummah*? Unfortunately, the annual observance of Ramadan shared by the Australian Muslim communities, or any other Western Muslim community for that matter, is poorly represented in scholarship. Literature on Ramadan in the West tends to be exclusively focused on health issues such as the impact of intermittent fasting during Ramadan on patients with diabetes (e.g. Alharbi et al., 2017; Almansour et al., 2017; Peterson et al., 2012), on weight and body composition (Fernando et al., 2019) or on exercise (Stannard & Thompson, 2007). An exception to these more health-related articles is the research of Piela and Krotofil (2021) and Jones-Ahmed (2022) on the impact of COVID-19 and the lockdown on adherents during Ramadan. They argue that Muslims have experienced Ramadan during the pandemic in a variety of ways—some saw the benefit of isolation as a means to reflect and connect more with God, while other felt the loss of social interactions provided by mosques and the community during this special period. Moreover, to date, no research has analysed how Ramadan is represented in Western media more broadly and Australian media specifically. As such, this article aims at considering Ramadan from a social scientific perspective and at how it is understood in a Western country such as Australia. It uses the region of Greater Western Sydney in New South Wales, Australia, as a case study of how Ramadan is represented in local and state print media and then considers how images of this important religious festival are curated on the Facebook sites run by local mosques.

This article focuses on two mainstream Sydney newspapers, *The Sydney Morning Herald* owned by Nine media and Murdoch owned *The Daily Telegraph*, first to capture the way journalists write about this religious festivity in one of Australia’s most multicultural cities. The news media, as Weng and Halafoff (2020) remind us, remains a primary source of information about Muslims for non-Muslims and has been a prominent vehicle through which negative stereotypes of Muslims and Islam are communicated in the public domain. This became especially problematic due to anti-Islamic sentiments after 9/11 (e.g. Jaspal & Cinnirella, 2019; Munnik, 2018), and there are elements of such stereotyping of Muslim Australians that remain so today (Weng & Mansouri, 2021). Weng and Wake (2021) argue that this is largely due to an Anglo-Celtic dominance in the media workforce. In Australia, where our research takes place, there is a ‘hidden religiosity’ and more specifically a ‘hidden Christianity’ in news reports, and when Islam is covered in the media, it is often done so in a sensationalised way (Weng & Halafoff, 2020; Weng & Mansouri, 2021). There has also been research on more media-focused Muslim topics such as mosques (e.g. Dunn, 2001; Vahed & Vahed, 2014) or *Shari’a* (Black & Sadiq, 2011; Possamai et al., 2013). These will be used below for comparative purpose with our research on the representation of Ramadan. This article then goes on to consider some key local newspapers from specific LGAs in GWS to understand the ways in which Ramadan may be perceived differently at a more local level. Using a visual ethnographic approach, it then compares the pictures used in these newspapers with those made publicly available by mosques in GWS in the representation of Ramadan. Through the lens of neo-liberal post-secularism and multiculturalism, this article argues that Ramadan is represented in print media as a welcomed festivity in Australia, thereby supporting a national narrative that celebrates ethnic and religious diversity, while simultaneously providing economic prosperity for the whole society. Indeed, Muslim adherents who are fasting are represented as vibrant valuable workers while generating economic activity during this festive time.

## Islam in Australia

Muslims are not new arrivals to Australian shores. Makasssan Muslims traded with Australian Aboriginal peoples and Torres Strait Islander peoples in the far north of the country in Arnhem land before the invasion of Christian colonisers (Bennett, 2006, 2014). Throughout Australia’s history, there has a strong presence of Muslims from early contact with Indonesian trepangers to Afghan cameleers and Malay pearlers in the mid-late 1800s. Although White Australia Policy (from 1901) limited the immigration of any persons who were not of *white* European heritage, in the 1920s and 1930s, *white* Albanian Muslims were granted entry to Australia followed by *white* Muslims from the Balkans in the aftermath of World War II. With the dismantling of these racist policies by the Whitlam government in 1973, Australia became a country of destination for waves of Muslims from across the world and for a myriad of reasons (such as for economic prosperity, to escape war, to re-unite with family members).^1^

According to the 2021 census, Muslims make up 3.2% of the Australian population, an increase of 35% of its previous population share of 2.6% reported in the previous census, 5 years earlier. The Australian Muslim population is incredibly diverse hailing from around 183 countries, but Sunni and Shia are the two largest denominations of Islam. While 37.2% are Australian born in the previous census (Hassan, 2018), other top countries of origin are Pakistan (9.3%), Afghanistan (7.2%), Lebanon (5.8%) and Bangladesh (5.7%). At the meso level in 2021, 43% of the whole Australia Muslim population live in NSW and 34% in Victoria.

Possamai and Tittensor (2022) analysed the proportion of Christian and non-Christian religions, and no religion, across Australian states. They discovered from 2016 census data that New South Wales (NSW) is simultaneously the most religiously diverse state in Australia, and highly Christian. Furthermore, Stevenson et al. (2010) mapped religious identification in Sydney local government areas (LGA) and discovered that this metropolis is more religious than the rest of the country. This is mainly caused by its patterns of non-Christian immigration, even though Christianity has overall decreased and the ‘No Religion’ category has grown. Stevenson et al. (2010) argue that Sydney is more a post-Christian city even if NSW remains among the most Christian states in Australia. Further to this study, Greater Western Sydney (GWS), a region that covers 13 LGAs and which is the third largest economy behind Sydney CBD and Melbourne CBD, is the location with the highest proportion of Muslim in Australia according to the 2016 census (9.1% for GWS, 5.3% for Sydney and 3.6% for NSW); that is an increase of 66.3% over a 10-year period between 2006 and 2016 (Lawton, 2016). As such, GWS is one of the fastest growing population in Australia and one of increasing cultural and linguistic diversity. Further, the majority (67%) of the mosques in NSW are located in the Western suburbs (Underabi, 2014). It is, thus, an ideal case study to understand Muslims in Australia and especially the celebration of Ramadan.

## Ramadan in newspapers

Newspaper articles on Sydney Ramadan celebrations from the *Sydney Morning Herald* (SMH) (*n* = 62), a centrist newspaper covering Sydney since 1831 and the most read paper in the country, were analysed to get a sense of how this important month in the Muslim calendar is portrayed in a mainstream media outlet. The article only considers Ramadan festivities in Sydney and its Western suburbs and thus excluded any articles that made reference to Ramadan celebrations overseas. We used ‘Ramadan’ as a keyword in the SMH’s digital archive to search articles for the period between 2011 and 2021 (10 years). Through an inductive approach, two of the authors, in regular consultation and discussion with the other contributors, then coded the results thematically. As these themes are ideal types, some articles below can sometimes be used more than once as they fit various categories.

Overall, the articles portray Ramadan as a welcomed event in a country that has been dominated by Christianity for over two centuries. We are far from the negative representations as found in the research on Muslim in the media discussed above. However, the way this festival is represented underscores a specific type of neo-liberal economic discourse that we are now further exploring.

One key finding is that Ramadan is showed to be a profitable period (*n* = 9) for businesses that cater to the Muslim market, especially food outlets that run all night for the entire holy month. The daily fasting ritual is often broken communally, and it is commonplace that this takes place in local eateries. The reports note an increase in sales for Halal butchers, clothing business and Hajj pilgrimage travel packages. As these two pre-pandemic articles mention:

Retail trade might be down elsewhere in Sydney but business is booming at Centro Bankstown as the Muslim community gears up for Eid al-Fitr, the three-day celebration that marks the end of Ramadan and traditionally a time to splash out on new outfits. (McKenny, 2011)

Even travel agencies are busy, offering package tours of a pilgrimage to Mecca. (Kembrey, 2014)

The focus on the commercial aspects of Ramadan can be understood by considering the way *Shari’a* (law) has been portrayed in the media. *Shari’a* is a way of life and is practised as part of a Muslim’s daily adherence. Discussions in the West about allowing *Shari’a* courts alongside common law have often become heated (Possamai et al., 2015). In Western countries, many populist politicians and journalists have used this as a claim that Muslim people are changing ‘their’ country. These debates are often sensationalist and overlook the fact that Muslims are obliged to follow the laws of their host country (for the first generation) and country of birth (for subsequent generations). These debates are often used to racialise Muslims Others. In practice, *Shari’a* law is often used adjacent to local laws. As Black and Sadiq (2011) and Possamai et al. (2013) have argued, if *Shari’a* challenges the Australian family and criminal laws, it is seen as ‘bad’, whereas if it deals with profit accumulation, it is seen as ‘good’. Indeed, when newspapers deal with Islamic finance and *Shari’a* compliant businesses, and the ability to make Australia more financially prosperous, it is seen in a positive light. While the overall Ramadan is far from being portrayed in a negative light compared to *Shari’a*, it is seen as positive when it is an asset to the Australian economy. Indeed, it feeds the national narrative of Australia embracing celebrating cultural diversity ‘multicultural’ narrative and is capable of boosting its neo-liberal economy.

Another key theme within the sample are articles focused on Muslim athletes who speak openly about their religion and difficultly fasting while playing high-level sports (*n* = 12). They show not only a dedication to their faith but also demonstrate their ability to be pragmatic in an Australian society that does not stop for this holiday. Although Ramadan is celebrated in this country, and in contrast to, for example, Christmas, it is not a time period to slow down. An example is:

They’re the women’s AFL team from western Sydney who have won six of their last seven games. Earlier this month they played the curtain-raiser at the MCG. And with about two-thirds of the team from a Muslim background, they’re not letting Ramadan slow them down. (Bullen, 2015)

This is aligned with Ramadan being a profitable venture as the economy of Australia is portrayed as not needing to stop for fasting, as backed up by these athletes with strong symbolic capital. This is particularly true in a country that is well known for its love of professional and past-time sport. Islam is thus represented as compatible with an ‘Aussie’way of life, and Muslim athletes are even praised for their commitment to their sports even during challenging fasting periods.

From a political aspect, many articles (*n* = 12) report the visit of Australian politicians to various functions during Ramadan such as the then PM Abbott speaking at an Iftar dinner (Robertson, 2013) or Turnbull hosting a dinner at Kirribilli House during Ramadan (Wyndham, 2016). This reflects the importance for the government of actively engaging with this religious event and its Muslim population thereby strengthening the ‘Australia as an inclusive nation’ narrative. This, however, does not always happen without any tension. For instance, in 2015, Muslim leaders boycotted the Iftar dinner prepared by the Australian Federal Police as a form of protest against the then Abbot government and its policing tactics targeting Muslim Australians (Olding, 2015; see Tittensor et al., 2020).

Although in this newspaper no article is critical of this now established religious celebration, some (*n* = 5) make unnecessary reference to Ramadan when reporting on criminal activities. Ramadan is used as a temporal stamp for cases involving an armed robbery and a missing child and also as a maker that these incidents invoked the Muslim Other.

As the crimes have no affiliation with Ramadan practices or celebration, this is illustrative of the continued stereotyping of Muslim Australians, particularly since the 9/11 attacks on the World Trade Centres. This process constructs Muslims as a homogenous group that ignores the group’s religious and cultural difference. A recent report from the Australian Human Rights Commission (2021) gave voice to Muslims in Australia who reported that the media’s portrayal of their religious community was sometimes unfair and inflammatory. The community leaders who participated in this study reported their frustration when trying to promote good stories within the media. Ramadan is such a story and even if overall positively well represented in *The Sydney Morning Herald*, the 5 articles in this category are unconsciously or discreetly part of an unbalance type of reporting.

Some recent articles (*n* = 10) covered how this religious festivity had to adapt to COVID-19 pandemic restrictions. They include reports on how Imams managed the challenge of empty mosques, the use of video calling technology at Iftar dinners and the community’s call to stay ‘positive’ during such difficult times. While an article by Mazzoni (2020) in the *Daily Mail* in the state of Victoria, made reference to a COVID cluster linked to Eid celebration in the region, none of these SMH articles raised the possibility of this celebration being linked to virus spreading events. Overall, these articles portray Muslims in Sydney as law-abiding citizen that followed pandemic mandates during their time of fasting, albeit at the expense of their usual social festivities.

Although the Muslims live across NSW, there are some localities with larger communities that reflect earlier settlement patterns. As such from these SMH articles, Lakemba is often the focus of articles on Ramadan (*n* = 11). This does not come out as a surprise as Lakemba, a suburb about 12 km from Sydney’s central business district, hosts the largest mosque in Australia. It is renowned for its annual night markets during the month of Ramadan. These markets include food stalls, music, cultural activities and outdoor settings. Other places in NSW with a high Muslim population are sometimes referenced in articles but not at the same rate (e.g. 2 for Auburn and Bankstown and 3 for Parramatta). These variations across LGAs are the focus of the next section of this article.

We tested these themes with another newspaper during the same time period, the *Daily Telegraph* (*n* = 25), a tabloid newspaper published in Sydney as well and by News Corp Australia. We did discovered similarities with the themes explored above (business, *n* = 9; sport, *n* = 1; COVID-19, *n* = 1; links with politics, *n* = 2; and Lakemba as well being the most locally focused part of Sydney, *n* = 9; see Table 1). Of interest were 3 articles reporting complaints about loud noises, extended hours of night celebrations and parking issues due to Ramadan (e.g. Beech, 2014). This was not reported on in the more centrist paper above.

BANKSTOWN Council will investigate the idea of implementing a licensing system for businesses that extend their trading hours during Ramadan, including fees.

[A councillor] told the council during last Tuesday’s meeting many Greenacre and Punchbowl residents had complained to him about noise, rubbish and health issues during the Islamic month of fasting, which ended yesterday (Beech, 2014)

**Table 1:** Ramadan in Sydney newspapers

	SMH	Daily Telegraph	Canterbury Bankstown Express	The Bansktown-Canterbury Torch	Mt Druitt-St Marys Standard	Blacktown Advocate
Business	9 (15%)	9 (36%)	13 (65%)	2 (17%)	0	0
Sports	12 (19%)	1 (4%)	0	0	0	0
Political	12 (19%)	2 (8%)	3 (15%)	5 (42%)	0	0
Covid	10 (16%)	1 (4%)	0	0	0	0
Link to criminality	5 (8%)		0	1 (8%)	0	0
Opposition to event		3 (12%)	4 (20%)	0	0	0
Total in Sydney/LGA	62	25	20	12	1	2

Although we do not see here vociferous oppositions and use of stereotypes as in the 1980s and 1990s with regard to opposition to mosque developments in Sydney (Dunn, 2001), we do observe that these comments might be a way to problematise multiculturalism in Australia. As Vahed and Vahed (2014) note with regard to opposition against the planning of new mosques in Australia, the language used is no longer about the character of Muslims and of their religion, but pivots towards the logistical problems (such as traffic congestion, parking problems and noise). The same narrative devices are applied to the troublesome logistics of accommodating Ramadan.

Overall, these articles reflect how Ramadan is *represented* as an example of the success and celebration of Australian everyday multiculturalism, as long as the month of Ramadan does not disturb that ‘common’ Australian way of life (as exemplified by the articles on successful sports people able to succeed in both cultures and those about the noise issues) and when festivities contribute to the economy.

Indeed, the media narratives does largely align with the work of Dunn et al. (2015) who argue that the majority of Australian Muslims live a life characterised by ‘ordinariness’ (that is, their concerns and desires were every day and mundane) and that their high level of religiosity did not preclude them from feeling a sense of belonging in Australia. Dunn et al (2015) did go on to note that these Australians did experience high incidents of racism, but that this was not associated with indicators of disaffection. It must be noted, however, that as Dunn et al. (2015) point out, the experience of racism by Sydney Muslims generated an expected wariness about the relations between non-Muslims and Muslims and is associated with a critical stance in the media (more generally). Moreover, Islamophobia cannot be universalised given its varied manifestations (see Dunn et al., 2021) Certainly, although everyday multiculturalism (see Wise & Velayutham, 2009), especially in a country such as Australia with a history of colonialism and white supremacy, is largely represented in a positive light, there remains racialised commentary that speak to the conditional acceptance of minorities.

## Ramadan in local NSW papers

While the newspapers above tend to cover Sydney as a whole region, this section will now explore local newspapers to compare smaller community-based representations of Ramadan. As the Muslim population is not homogenous with concentrations of certain communities spread geographically, we considered that these local newspapers would focus on more specific sub-groups. In total, there are 12 LGAs in GWS, and we focused on a sample of four LGAs.^2^

Both the *Canterbury Bankstown Express* and the *Bansktown-Canterbury Torch* cover Bankstown (31.1% of Muslims) and Lakemba (61.1% of Muslims), both located in the Canterbury-Bankstown LGA (23.6% of Muslims). In the *Canterbury Bankstown Express*, we find again a high proportion of this event represented as a business (65%; see Table 1), but also 4 articles (20%) on noise complaints connected to the Ramadan night markets. This local newspaper is also affiliated with the *Daily Telegraph* where these articles seem to have filtered at the state level. Articles are focused on Ramadan celebrations taking place in its LGA especially at Lakemba (*n* = 13). In *The Torch*, there are less articles on Ramadan, and all articles are based in Lakemba.

*The Blacktown Advocate* and the *Mt Druitt-St Marys Standard* cover the Blacktown LGA (8% of Muslims) where Rooty Hill (12.4% of Muslims) is located. Despite the presence of a mosque at Rooty Hill, there are only 3 articles with a local focus in total for these Blacktown newspapers. The 2 from the *Blacktown Advocate* are mainly about hazard to the health of diabetic patients fasting during Ramadan, and the one from *Mt Druitt-St Marys Standard* focuses on the Ahmadiyya Muslim community.

Two LGAs in NSW however have a checkered history with the treatment of Muslims. The first is the Sutherland Shire (about 25 km south of Sydney’s CBD) where in 2005, tension between the predominantly white residents of the beachside suburb Cronulla and beachgoers of “middle eastern appearance” erupted in violent race riots where these beachgoers where attacked by a crowd of 5000 white Australian men (see Noble, 2009). In 2008, the LGA of Camden was host to a protest by up to 1000 people against the building of a private Islamic school, which was followed by a text message campaign condemning the school’s erection (Al-Natour & Morgan, 2012).

The most noticeable finding from Camden’s local newspapers (i.e. *Camden-Narellan Advertiser*, the *District Reporter* and *Oran Park Gazette*) is that Ramadan is a “outside” celebration. Articles tend to be about the event *outside* of Australia, usually in relation to human conflict (e.g. Israeli and Palestinian conflict in Jerusalem or the Taliban’s violence continuing during this ‘so-called’ month of peace and festivities). There are no articles about the celebration of this religious event from within this LGA. If there are references to Sydney, it is in other LGAs such as Campbelltown, the ‘next door’ and more ethnically diversified LGAs, even if the Camden LGA has 4.8% of Muslims from its overall population. Cleary, this festivity is a ‘foreign’ event to this locality—it is celebrated by the distant Other. Similar findings were discovered for the Sutherland Shire with *The Leader* where the Muslim population is even smaller (0.9%).

From this analysis, it appears that newspaper are more likely to report local events about Ramadan where there are public activities (e.g. food stalls, children entertainment and other fun activities) rather than mosque activities or home prayers and devotion by its Muslim population. These public events, even if happening in a religious context, are reported as if they were secular in nature.

## Visual ethnography of pictures of Ramadan in Greater Western Sydney

To push the analysis further, a visual ethnography of the photos provided in the newspapers above was contrasted with photos provided by the Muslim community itself during the same time period. We used a netnography and visual ethnography to examine Ramadan through its digital exposure on the Internet in Greater Western Sydney. Netnography combines ethnography and the Internet by observing aspects of the Internet which include image and textual data (Costello et al., 2017). Visual ethnography is the analysis of visual materials that aim to interpret and understand parts of a culture deeply (Pauwels & Mannay, 2020). For this, we used samples of public pictures on Ramadan provided to the public at large by three mosques (the Guildford mosque catering mostly to the Turkish community, and the Bankstown and Rooty Hill mosques catering mostly to the Pakistani community). Not all mosques provided pictures of their Ramadan celebrations every year.

The overall first observation is that these mosques, with the exception of Rooty Hill mosque showing Muslims of African appearance in some of its images, tend to report pictures about their own ethnic community, which are contrasted in the newspaper articles showing people from different cultural backgrounds coming together for Ramadan. This reflects the fact that Australian mosques tend to be ethnically organised (Bouma, 1994; Underabi, 2014) and that there is correlation between the ethnicity of the imam, the members of the management committee and the ethnicity of the attendants.

While the pictures of the mosque show the religious aspect of this festivity, the newspapers articles deal more with the cultural, social and commercial aspect of this event as lived in the everyday life of Muslims in Sydney. With this religious depiction from insiders, we can observe a quiet and solemn celebration, whereas those from newspapers show a vibrant and colourful celebration.

What strikes the viewer of pictures of community engagement through the Iftar nights and other events is that it is mostly men’s spaces that are represented on these insider’s pictures, as women are mostly absent from these pictures. Bankstown Masjid (*n* = 68) does not have any photo of adult women. There are few with young girls, mixed with men during Iftar. Rooty Hill Masjid (*n* = 179) does not show any female or mixed spaces. Guildford mosque (*n* = 431), on the other hand, shows photos of men and women in the same room but seated separately. None, however, shows a woman’s space. While adult and young women are underrepresented in these pictures, men from all ages are showed to be participating in the Ramadan events. These reflect a traditional representation of Ramadan that maintains gender segregation. This is a contrast to the pictures analysed in newspapers where women are far more visible celebrating Ramadan.

Various Muslim associations, Islamic centres and welfare charities (*n* = 47) were contacted in January 2022 asking them to provide us with pictures of the way they celebrate Ramadan. Some did not answer, others stated they did not have pictures related to Ramadan, or that they did not want to share their pictures as some of their members are recognisable (even if we explained that we will keep any information anonymised as approved by our university ethics committee). Among these, only two replied with consideration towards providing these pictures. One directed us to their Facebook page which uses Persian and is Shi’a. The analysis showed a similarity with the mosques above. From the 291 pictures of people taken during Ramadan, 234 were on male spaces and 57 on mixed spaces, but none of female spaces. For most of the mixed spaces, women tend to be mostly obscured in the back or on the side. No pictures of girls could be found on any of these pictures.

Another finding of interest is the representation of technology. Images used prior to COVID-19 show an ad hoc usage of technology during Ramadan, such as an electronic Ramadan calendar or the use of mobile applications to note the Iftar timings. As COVID-19 affected religions, mosques had to adapt themselves and started to use online livestreams and live transmissions through mainly Facebook and YouTube during these festivities. This is also reflected in pictures from the newspapers above discussing Ramadan and COVID-19, in which we can observe a strong surge of the use of online technologies during the pandemic. This is not different from other religions that had to adapt to this new environment (e.g. Rocha et al., 2021; Campbell & Osteen, 2021), but what is of importance for this research is that in these representations, the use of these online way of delivering the religious message has been normalised and become part of everyday life.

From this analysis, it becomes clear that the way Ramadan is represented in newspapers is more about its secular and celebratory aspects, whereas from an inside perspective, it is more about devotion and its religious aspect. In these ‘secular’ depictions, we see a stronger mix of ethnicities (i.e. intercultural) and gender, whereas on the ‘religious’ one, we can observe stronger segregations and intra-culturalism.

## Neo-Liberal Post-Secularism and Multiculturalism

Post-secularism is a concept used to analyse the re-emergence of religion in the public sphere and how these religious groups, including atheism, can co-exist together in a positive way. Religion has re-entered the public sphere and in the case of this article is contributing to neo-liberal representations of multicultural Australia (see, for example, Gauthier et al. (2013) and Burchardt (2017)). Not all countries have been secularised or post-secularised the same way (see Possamai & Tittensor, 2022), and each case can be different. Indeed, as the type of secularism is different between France and the USA, its path towards post-secularism will also be different. Possamai (2017) analysed the type of post-secularism that Australia is specifically experiencing and argued that in fact, post-secularism reflects a specific type of low key secularism that is in confluence with neo-liberalism. Ramadan, as portrayed in newspapers, does fit with this description. While not officially recognised as a public holiday as in the case of Christian Christmas or Easter, arguably due to the persistence of colonial-settler Christian hegemony, this festivity is overall supported, especially if it has the potential to lead to economic prosperity for certain groups and companies, and especially if it does not prevent the Australian way of economic life.

The way that Islam can help the growth of the economy is not limited to Ramadan. Nasir (2016) argues that Muslim consumer market and ‘halal’ products businesses (such as non-alcoholic toiletries) have recently grown extensively. In 2008, the global halal market was worth US $2.1 trillion. Halal tourism was worth US $128 billion in 2014, and this excludes hajj (Shirazi, 2016: 12). Narrowing these observations to a national market, the Muslim Council of Britain (www.mcb.org.uk) presents itself as representing over 500 organizations, mosques, charities and schools in Britain. It recently issued a report titled ‘The Muslim Pound’ to highlight how Muslims can contribute to an economy in a Western country, as well as to empower young Muslim entrepreneurs. The report states that ‘in addition to contributing Britain’s economic vitality, the British Muslim community has created new drivers for growth due to the requirements of our faith’ (Muslim Council of Britain, 2013: 9).

Shirazi (2016) highlights a new targeted marketing specifically aimed at Muslims, and involving, among other niche products, children’s toys (e.g. burqa dolls) and games promoting Islamic values (e.g. the Mecca to Medina board game). As an overview of this phenomenon, she (2016) refers to this new marketing strategy as ‘Brand Islam’. In her analysis, she also addresses the facts that customers show consumer loyalty and that this is a growing trend. Here Islam is being commodified and the Muslim economy is part of an expansion of markets and neo-liberal ideology.

This type of post-secularism in affinity with the economy coincides with the emergence of neo-liberal multiculturalism (Kymlicka, 2013; Roose & Possamai, 2015) in which ethnic immigrant entrepreneurships, transnational commercial linkages and remittances are highly valued for the economy. While the first-wave of neo-liberals were critical of multicultural policies in countries such as Australia, the current wave sees its potential of integrating the minorities of one’s country into global markets. This is indeed reflected in the way Ramadan is portrayed in the media where its business aspect is highlighted. The silence in neo-liberal multiculturalism on economic redistribution, racial inequality, unemployment and labour rights is also in sotto voce in these articles.

## Conclusion

The analysis of the reporting of Ramadan in Sydney newspapers has revealed that this religious festivity tends to be reported in a secular fashion with a stronger focus on its public and festive activities. The focus of a large proportion of these articles on the way it attracts business demonstrates that it is a well-accepted event in Australia’s neo-liberal post-secularism and multiculturalism. The fact that sports people are recognised for being able to continue their work while fasting is also a symbolic message that workers should not stop and can remain productive despite their religious observance. It also indicates their ‘loyalty’ to the sport obsessed Aussie way of life. These articles did not show much antagonism against this festivity except when it comes to public disturbance. There was no undermining of its Muslim characteristic. The local papers followed a similar trend, but the articles tend to be more about these public festivities than about the celebration of local mosque or of its local Muslims citizens.

In contrast, the pictures provided in Muslim sites in Sydney are more religious than the newspaper depiction and show a contrast with regard to ethnicity and gender; they are more intra-cultural and traditional. While the newspaper pictures are from the public sphere and tend to be multicultural across Muslim various ethnicities and do not show gender segregation, the online pictures from Muslim organisation show a strong gender segregation and a disengagement from multi-ethnicity. The public portrayal of Ramadan is in this context portrayed as festivities good for the economy, multiculturalism and gender equality, whereas the more domesticated one are more religious, focused on one ethnicity and male dominant. While the representations in the public sphere are part of neo-liberal post-secularism and multiculturalism, those from these organisations are more religious and traditional. Further research on this topic would benefit from working on an international comparison with other global cities.

## Data Availability

Not applicable.

## Footnotes

1 For a detailed history of Muslims in Australia, see Kabir (2005) and Haveric (2019).

2 The statistics provided below are from the 2021 Census as reported on the Australian Bureau of Statistics site (www.abs.gov.au).
